# Triazine Herbicides Risk Management Strategies on Environmental and Human Health Aspects Using In-Silico Methods

**DOI:** 10.3390/ijms24065691

**Published:** 2023-03-16

**Authors:** Tianfu Yao, Peixuan Sun, Wenjin Zhao

**Affiliations:** College of New Energy and Environment, Jilin University, Changchun 130012, China

**Keywords:** triazine herbicides, cleaner production, 3D-QSAR, molecular docking, molecular dynamics, microbial degradation pathways, field application program

## Abstract

As an effective herbicide, 1, 3, 5-Triazine herbicides (S-THs) are used widely in the pesticide market. However, due to their chemical properties, S-THs severely threaten the environment and human health (e.g., human lung cytotoxicity). In this study, molecular docking, Analytic Hierarchy Process—Technique for Order Preference by Similarity to the Ideal Solution (AHP-TOPSIS), and a three-dimensional quantitative structure-active relationship (3D-QSAR) model were used to design S-TH substitutes with high herbicidal functionality, high microbial degradability, and low human lung cytotoxicity. We discovered a substitute, Derivative-5, with excellent overall performance. Furthermore, Taguchi orthogonal experiments, full factorial design of experiments, and the molecular dynamics method were used to identify three chemicals (namely, the coexistence of aspartic acid, alanine, and glycine) that could promote the degradation of S-THs in maize cropping fields. Finally, density functional theory (DFT), Estimation Programs Interface (EPI), pharmacokinetic, and toxicokinetic methods were used to further verify the high microbial degradability, favorable aquatic environment, and human health friendliness of Derivative 5. This study provided a new direction for further optimizations of novel pesticide chemicals.

## 1. Introduction

Triazine herbicides (THs) have long held an important position in the pesticide market. They are applied primarily to maize cropping fields [1], owing to their broad spectrum, high performance, and low cost [1], but they also have high toxicity, environmental persistence, and endocrine disrupting effect [1,2]. THs target the D1 protein (D1-PSII) of the photosynthetic system II (PSII) and act as herbicides by inhibiting plant photosynthesis [3]. However, only 10–30% of THs are currently absorbed by target plants or adsorbed by soil particles, with the majority polluting water bodies, such as surface water, via surface runoff and irrigation, eventually reaching the ocean [4]. In 2020, the US Environmental Protection Agency (EPA) designated atrazine (ATZ), promazine, and simazine as pesticides “likely to adversely affect (LAA)” in species and ecosystems [5]. Furthermore, the introduction of bans or restrictions on the use of 1, 3, 5-Triazine herbicides (S-THs) in the EU, Ulaanbaatar, Nigeria, and India highlights the continued residues and biohazards of S-THs in the environment [6]. The continuing residues and biohazards of S-THs in the environment have attracted widespread attention.

S-THs are highly persistent in soil and aqueous sediment environments, with half-lives of 4–12 weeks for prometryne (PRT) [7] and 4–57 weeks for ATZ in soil environments, respectively [8]. In addition, S-THs can pollute the aquatic environments through rainfall, irrigation, and surface runoff, causing long-term damage to aquatic organisms. For example, S-THs are acutely toxic to fish, and 1200 μg/L PRT can significantly reduce embryo hatching and survival in carp [9]. Hao et al. [10] discovered that ATZ reduced zebrafish hatching rates and increased the incidence of malformations and embryo mortality with increasing ATZ exposure, which also caused necrosis and congestion in carp gill epithelial cells [11], oxidative stress in catfish liver and gills [12], and ovarian lesions in female blackhead dull fish [13]. Furthermore, S-THs can be harmful to humans, with PRT being toxic to human pulmonary adenocarcinoma cell lines and human bronchial epithelial cell lines [14], and ATZ causing carcinogenesis, teratogenesis, and mutagenesis after long-term exposure [15,16].

Soil microorganisms such as *Acinetobacter* spp. Brisou and Prévot (Moraxellales: Moraxellaceae), *Arthrobacter* sp. Conn and Dimmick (Micrococcales: Micrococcaceae), *Agrobacterium* sp. Conn (Hyphomicrobiales: Rhizobiaceae), *Bacillus* sp. Cohn (Bacillales: Bacillaceae), *Deinococcus* sp. Brooks and Murray (Deinococcales: Deinococcaceae), *Microbacterium* sp. Orla-Jensen (Micrococcales: Mycobacteriaceae), *Nocardioides* sp. Prauser (Propionibacteriales: Nocardioidaceae), and *Rhodococcus rhodochrous* Tsukamura (Mycobacteriales: Nocardiaceae), among others, have been shown to degrade S-THs partially [17,18,19,20,21,22,23,24]. White-rot fungi (*Phanerochaete chrysosporium* Burds. (Polyporales: Phanerochaetaceae)) and lignocellulose-degrading fungi (*Pleurotus pulmonarius* Fr. (Agaricales: Pleurotaceae)) can dechlorinate ATZ water to produce hydroxylated ATZ and dealkylated ATZ metabolites with nitrogen [25,26]. Although various microorganisms can degrade S-THs, none of the triazine rings are broken, resulting in the persistence of S-THs in the soil. Vonberg et al. [27] showed that, although ATZ has been banned in Germany for 31 years, residue can still be detected in groundwater, surface water, and soil. Therefore, developing functional and environmentally friendly substitutes for S-THs is crucial for the ecological environment and human health.

The three main research objectives of this study are as follows: (1) create S-THs substitutes (with high herbicidal functionality, high microbial degradability, and high environmental friendliness but low human lung cytotoxicity). (2) to design and screen optimal field application schemes (with high microbial degradation promotion in maize cropping fields. (3) to further verify and assess the excellent comprehensive performance of S-THs substitutes and their degradation products (with high microbial degradability, and favorable aquatic environment, and human health friendliness).

## 2. Results

### 2.1. Construction and Evaluation of the Single-Effect and Comprehensive-Effect 3D-QSAR Models of Herbicidal Functionality Properties, Microbial Degradability, and Human Lung Cytotoxicity of S-THs

The structural information of 26 S-THs was used as an independent variable, and the docking score (LibDock Score, LDS) values of herbicidal functionality properties, microbial degradability, and human lung cytotoxicity (hereafter referred to as herbicidal functionality properties, degradability, and toxicity) of S-THs were adopted as the dependent variables to build the single-effect CoMSIA models for herbicidal properties, degradability, and toxicity of S-THs (Table S1). In addition, the final weight results of herbicidal functionality properties, degradability, and toxicity of the comprehensive value (CV) are shown in Figure S1, and the results of the CV calculations are shown in Table S1. The CV was adopted as a dependent variable to construct the comprehensive-effect CoMSIA model of herbicidal functionality properties, degradability, and toxicity of S-THs. Table S2 lists the relevant modeling materials.

In addition, the evaluation parameters of the single-effect CoMSIA and comprehensive-effect CoMSIA models of herbicidal functionality properties, degradability, and toxicity of S-THs are shown in Table 1. The comprehensive-effect CoMSIA model of herbicidal functionality properties, degradability, and toxicity of S-THs was used as an example; the model cross-validation coefficient q^2^ was 0.751 (>0.5), the best principal component n was 10, the non-cross-validation coefficient R^2^ was 0.998, and the standard deviation was 0.008, manifesting that the constructed model had an excellent internal prediction and fitting ability [28]. In addition, the model test set external validation interaction test coefficient r^2^_pred_ was 0.678 (>0.6), manifesting that the constructed model had favorable external prediction ability [29]. The model (R^2^ − q^2^)/R^2^ (<30%) manifested that the constructed model was not over-fitted [30].

### 2.2. Design of S-TH Substitutes Based on the 3D Isopotential Diagrams of the CoMSIA Model

ATZ, which is primarily used in agriculture, was chosen as the target molecule to analyze the three-dimensional (3D) isopotential diagrams of single-effect and comprehensive-effect CoMSIA models of herbicidal functionality properties, degradability, and toxicity and to design the substitutes. Figure S2 depicts the molecular structure and proposed modification sites for ATZ.

**Figure 1:**
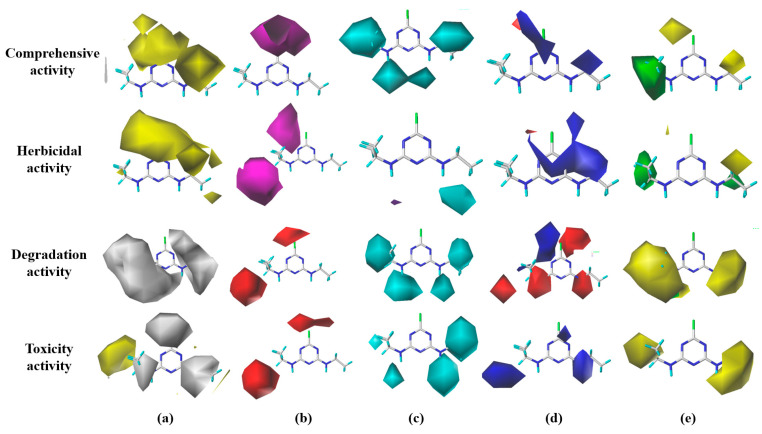
The 3D isopotential diagrams of the single-effect and comprehensive-effect CoMSIA models of herbicidal functionality properties, degradability, and toxicity of S-THs: (**a**) hydrophobic field; (**b**) hydrogen-bonded acceptor field; (**c**) hydrogen-bonded donor field; (**d**) electrostatic field, and (**e**) steric field.

The 3D isopotential diagrams of the hydrophobic (H), hydrogen-bonded acceptor (A), hydrogen-bonded donor (D), electrostatic (E), and steric (S) fields for the single-effect and comprehensive-effect CoMSIA models of herbicidal functionality properties, degradability, and toxicity of S-THs are shown in Figure 1.

In this study, we aimed to reduce the cytotoxicity of S-THs in the human lung by designing ATZ substitutes based on the reverse law of the substitution principle of 3D isopotential diagrams. According to the single-effect and comprehensive-effect CoMSIA models of herbicidal functionality properties, degradability, and toxicity of S-THs, and the contribution ratio of each force field in the 3D isopotential diagrams (Table 2), single, double, and multiple substitutions could be performed at the 1–5 point sites of ATZ (Table S3).

Therefore, hydrophobic groups (-F, -Cl, -Br, -SH, -C≡C, -OCH_3_, and -CF_3_) were introduced to site 1, a more electronegative group (-CF_3_) was introduced to site 3, and a small volume of group (-CH_3_) was introduced to site 4, to design and screen a total of S-THs with improved single- and comprehensive-effects of S-TH substitutes (Table S3).

### 2.3. Prediction and Evaluation of the Single-Effect and Comprehensive-Effect 3D-QSAR Models of Herbicidal Properties, Microbial Degradability, and Human Lung Cytotoxicity of S-THs

In this study, the herbicidal functionality properties, degradability, and toxicity of the 40 designed substitutes were predicted using four constructed CoMSIA models, and the predicted values were normalized using Formula (2). The functionality and degradability (positive indices) were normalized by “bigger, better type,” while toxicity (negative indices) was normalized by “smaller, better type” (Table S4). The comprehensive effect of the 40 S-TH substitutes ranged from -26.79% to 70.44%, while the eight S-THs, D-3, D-4, D-5, D-18, D-29, D-30, D-31, and D-35, were consistent with the weighted values of the comprehensive-effect model (49.31%:25.17%:25.52%). The results verified the effectiveness of the comprehensive-effect CoMSIA model of herbicidal functionality properties, degradability, and toxicity of S-THs, the reasonableness of the molecular design of the substitutes, and verified that the hydrophobic, electrostatic, and steric fields of the comprehensive-effect CoMSIA model were the primary factors influencing the comprehensive effects of S-THs.

### 2.4. Evaluation of the Microbial Degradability Universality and Toxicity of Antioxidant Systems in Fish of S-TH Substitutes

#### 2.4.1. Evaluation of the Microbial Degradability Universality of S-TH Substitutes

We selected three other target proteins for the microbial degradation of S-THs in addition to triazine hydrolase (TrzN), namely AtzC (PDB ID: 2QT3), LiP (PDB ID: 1B85), and MnP (PDB ID: 1MNP) [31,32] using the Protein Data Bank (PDB) database [33]. Molecular docking of the S-TH substitutes with the above three proteins was carried out. The LDS was used as an evaluation index to assess the microbial degradability of the S-TH substitutes. It was found that the microbial degradability of the eight S-TH substitutes, including D-3, D-4, D-5, D-18, D-29, D-30, D-31, and D-35, improved to varying degrees compared to ATZ or remained essentially unchanged (Table S5).

#### 2.4.2. Evaluation of the Toxicity of Antioxidant Systems in Fish of S-TH Substitutes

We selected two antioxidant proteins from carp, SOD (UniProt ID: Q8JFG7) and CAT (UniProt ID: E2CWE8), using the UniProt database [34]. Eight S-TH substitutes previously screened were molecularly docked to the two antioxidant proteins. The LDS of the two proteins were added together using a 1:1 weighting, and the CV of the toxicity of the antioxidant system in fish of S-TH substitutes was calculated (Table S6). Compared to ATZ, the toxicity of five S-TH substitutes (D-4, D-5, D-19, D-21, and D-25) was lower in the antioxidant system (range 0.12–23.98%), with substitute D-5 showing a significant reduction.

### 2.5. Screen of Optimal Field Application Schemes to Promote the Microbial Degradation of S-TH Substitutes in Maize Cropping Fields

**Figure 2:**
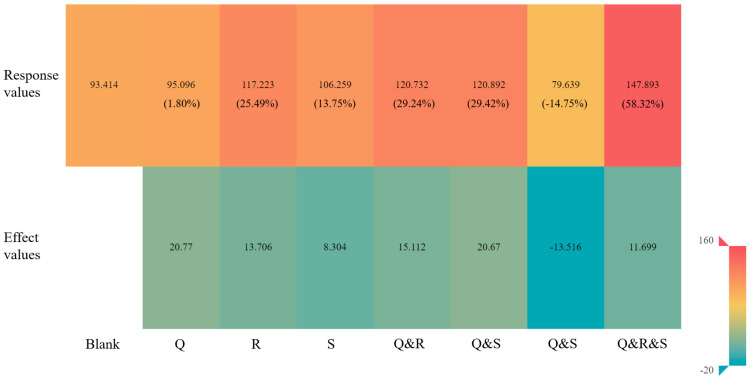
Response and effect values for the 8 external conditions schemes (3-factors/2-levels).

Compared to the blank control group (group 1, with a binding energy of −93.414 kJ/mol), the results of the Taguchi orthogonal experiment (Table S7) revealed that the binding energy values of groups 2, 10, 14, 15, 19, 21, 26, 27, 28, and 29 all showed varying degrees of reduction (1.51–58.32%), with group 2 (binding energy of −147.893 kJ/mol) showing the most significant reduction. Therefore, the external conditions from group 2 (aspartic acid (Q), alanine (R), and glycine (S)) were chosen as a field application scheme to perform a full factorial design experiment used with the 3-factors (Q, R, and S) and 2-levels (0 for no addition and 1 for addition), with a total of 8 different sets of external conditions schemes. The absolute values of the binding energy of the eight schemes were calculated and used as the response values for the factor analysis about the main-, second-, and third-order interaction effects among the three factors (Figure 2, Table S8).

The results of the main-, second-, and third-order interaction effects of each scheme (Figure 2) showed that the main-effect values of Q, R, and S (groups 2, 3, and 4) were positive, indicating that the main-effect factors (Q, R, and S) in the optimal field application schemes could promote the microbial degradation of S-TH substitutes in maize cropping fields. In the second-order interaction effects (groups 5, 6, and 7), the second-effect values of Q and R, and Q and S were positive, while the second-effect value of R and S was negative, indicating that the coexistence of aspartic acid and alanine, and aspartic acid and glycine exhibited synergistic effects in promoting the microbial degradation of S-TH substitutes in maize cropping fields. In contrast, the coexistence of alanine and glycine exhibited antagonistic effects. In the third-order interaction effects (group 8), the third-effect value of Q, R, and S was positive, indicating that aspartic acid inhibited the antagonistic effects of alanine and glutamic acid on the microbial degradation of S-TH substitutes, indicating significant synergistic effects of aspartic acid, alanine, and glycine. Consequently, combined with the maximum response value of Q, R, and S in Group 8 (value 147.893), the coexistence of Q, R, and S could be screened as the optimal field application schemes to promote the microbial degradation of S-TH substitutes in maize cropping fields.

### 2.6. Simulation of Microbial Degradation Pathways of S-TH Substitutes

**Figure 3:**
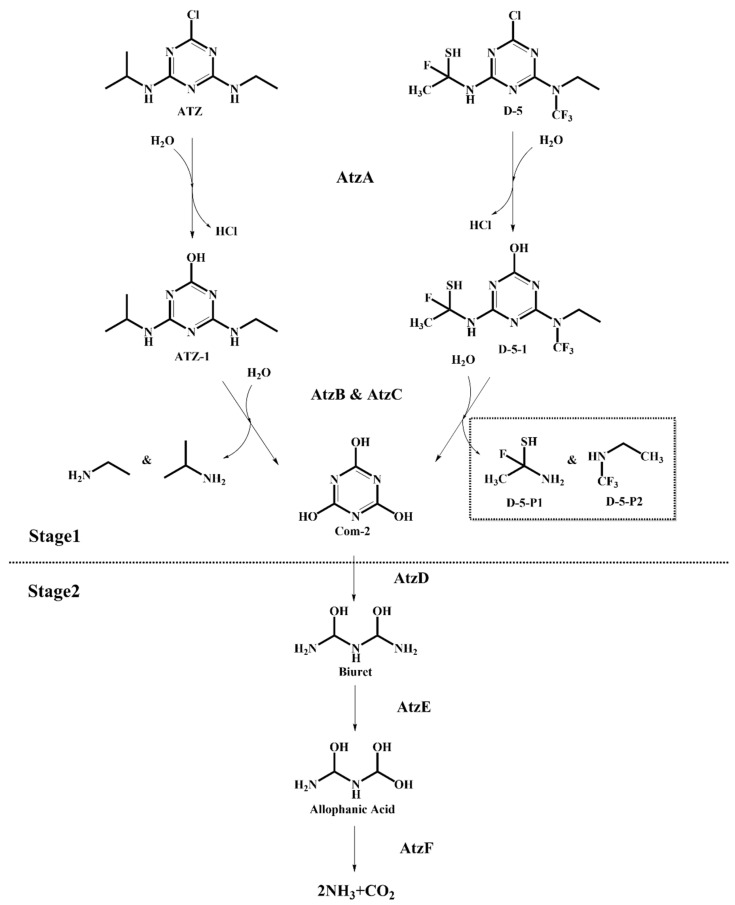
Simulation of microbial degradation pathways of ATZ and substitute D-5.

Figure 3 shows that the microbial degradation pathways of ATZ and substitute D-5 have the same process, Stage 2, with the only difference being the reactants and products of Stage 1. Therefore, the microbial degradation of ATZ and substitute D-5 was analyzed by comparing the differences of the two-step (Steps 1 and 2) reaction energy barrier (ΔE) in Stage 1 (Table 3).

It has been shown that the ΔE (>0) represents the difficulty of reaction occurrence, with smaller values indicating that the reaction is more likely to occur [35]. Compared to the ΔE values of Stage 1 (Steps 1 and 2) of the ATZ, that of the substitute D-5 were reduced by 62.62% and 13.85%, respectively, indicating that the groups on the modified S-THs triazine ring (-C_2_H_5_SNF, and -C_3_H_5_NF_3_) were more susceptible to be hydrolyzed to triuric acid by microorganisms. In addition, the above analysis results demonstrated that the microbial degradability of S-TH substitute D-5 was significantly enhanced, confirming the rationality of the comprehensive-effect CoMSIA model of herbicidal functionality properties, degradability, and toxicity of S-THs and the precision of the molecular design of substitutes constructed in this study.

### 2.7. Evaluation of Aquatic Biotoxicity and Human Health Risks of Microbial Degradation Products of S-TH Substitutes

As shown in Tables S9 and S10, regarding aquatic toxicity, the intermediate microbial degradation products D-5-P1 and D-5-P2 were significantly less toxic to green algae and fish and, to a lesser extent, Daphnia than ATZ. Regarding human health risks, compared to that of ATZ, the hepatotoxicity of D-5-P1 and D-5-P2 was significantly reduced, whereas the maximum tolerated dose was significantly increased. The carcinogenicity of D-5-P1 in male mice was reduced to nontoxic levels. The toxicity levels of the five toxicity models, including skin irritation, sensitization, and carcinogenicity, remained unchanged in male and female rats and female mice. The skin sensitization level of D-5-P2 was reduced to a low toxicity level, and the toxicity levels of the five toxicity models, including skin irritation and rodent carcinogenicity, remained unchanged.

## 3. Discussion

In the present work, the single-effect and comprehensive-effect CoMSIA models of herbicidal functionality properties, microbial degradability, and human lung cytotoxicity of S-THs were constructed, which showed excellent stability, predictability, and fitting, and the S-TH substitutes with excellent comprehensive performance were designed based on the 3D isopotential diagrams of the above models.

Overall, the hydrophobic field contributed the highest proportion to the molecular effect of S-THs in the four CoMSIA models, which was regarded as the main modifying force field to improve the herbicidal functionality properties of the S-TH substitutes. The single-effect CoMSIA model of herbicidal functionality properties contributed the highest proportion among the four models. The analysis of the 3D isopotential diagrams of the single-effect CoMSIA models of degradability and toxicity showed that the electrostatic fields have the largest and most comprehensive difference in the color block distribution, with the largest contribution of the E fields to the S-THs performance (17.8% and 19.4%, respectively). Therefore, the E field was regarded as the main modifying force field to improve the degradability of the S-TH substitutes and avoid increased toxicity. Furthermore, in the single-effect CoMSIA model of toxicity, the stereoscopic field accounted for the highest proportion, which was regarded as the main modifying force field to reduce the toxicity of substitutes for reverse design. Similar to our previous study, based on the constructed plant-microbial synergistic degradation CoMISA model of quinolones (QNs), the hydrophobic field and electrostatic field of this model were regarded as the main modifying force field. By introducing groups with hydrophobicity (-SH, -Cl, and -F), as well as groups with strong electronegativity (-CF_3_, -CH_3_, and -CH_2_F), QNs substitutes with enhanced plant-microbial synergistic degradation effect were designed reasonably [36].

In addition, the optimal field application scheme of aspartic acid, alanine, and glycine indicated the effective role of amino acids in the promotion of S-THs microbial degradation. Shen et al. [37] found that the stress of organic pollutants, such as petroleum hydrocarbons, could lead to a positive plant response and, to some extent, promote the secretion of amino acids in soil inter-roots. Furthermore, amino acid content had been correlated with soil N effectiveness, which improved the activity and respiration of soil microorganisms, and further enhanced the degradation of organic pollutants by soil inter-rooted microorganisms [38,39]. In addition, Li et al. [40] discovered that root secretions could effectively stimulate microbial degradation of organic pollutants within plant roots, which was consistent with the results of the present study that the coexistence of the three root secretions, aspartic acid, alanine, and glycine, could promote the microbial degradation of S-THs substitutes in maize cropping fields.

The simulation of microbial degradation pathways of S-TH substitutes indicated that, compared to the ΔE values of Stage 1 (Steps 1 and 2) of the ATZ, the substitute D-5 were reduced by 62.62% and 13.85%, respectively, indicating that the groups on the modified S-THs triazine ring (-C_2_H_5_SNF, and -C_3_H_5_NF_3_) were more susceptible to be hydrolyzed to triuric acid by microorganisms. In addition, the above analysis results demonstrated that the microbial degradability of substitute D-5 was significantly enhanced, confirming the rationality of the comprehensive-effect CoMSIA model of herbicidal functionality properties, degradability and toxicity of S-THs, and the precision of the molecular design of substitutes constructed in this study. Furthermore, Fu et al. [41], Li et al. [42], and Xue et al. [43] all used pharmacokinetic and toxicokinetic methods to predict and evaluate the human health risks of designed substitutes. The predicted results of indictors (hepatotoxicity, maximum tolerated dosage, skin sensitization, skin irritation, and rodent carcinogenicity) all indicated that the designed molecules had a low risk to human health. According to the above studies, the human health risk assessment based on pharmacokinetic and toxicokinetic methods had certain rationality and reliability. Therefore, the aquatic biotoxicity and human health risks of the microbial degradation products of the substitute D-5 designed in the present work were significantly reduced.

The in-silico methods used in this study have certain efficiency, rationality, and convenience, which could provide a new direction for the research and development of more similar functional chemicals. However, in the future, we still need to combine as much experimental data as possible. The environmental and human health hazards, as well as economic applicability and other aspects, should be taken into more comprehensive consideration in order to obtain more efficient and environmentally friendly new chemical substitutes.

## 4. Materials and Methods

### 4.1. Characterization of Herbicidal Functionality Properties, Microbial Degradability, and Human Lung Cytotoxicity of S-THs—Molecular Docking Method

This study selected 26 S-THs, including ATZ. First, the molecular structures were drawn and optimized using the Sketch Molecule, Minimize and Align Database modules of SYBYL-X2.0 software (Tripos, Inc.: St. Louis, MO, USA). Then, the molecular structures were optimized using Tripos force fields, Gasteiger-Huckel charges, and 10,000 iterations [44] to achieve the optimal conformation with the lowest molecular energy.

**Figure 4:**
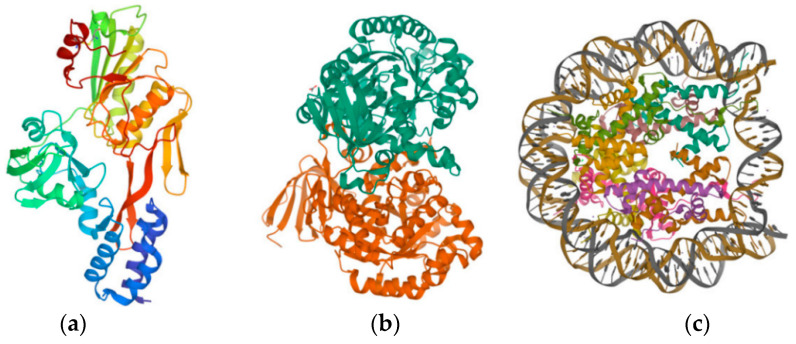
Schematic structure of the herbicidal, degradative, and toxic receptor proteins of S-THs: (**a**) 1FC9; (**b**) 4L9X; (**c**) 6K1J.

The PDB database [33] was used to identify and select the three target proteins mentioned above (Figure 4): the D1 protein of *Tetradesmus obliquus* Turpin (Sphaeropleales: Scenedesmaceae) photosynthetic system II (D1-PSII, PDB ID:1FC9) [45], the triazine hydrolyzable protein of *Paenarthrobacter aurescens* Phillips (Micrococcales: Micrococcaceae) (TrzN, PDB ID:4L9X) [46], and the human H2AX protein (H2AX, PDB ID:6K1J) [47] (the rationale for the selection of the above three proteins is shown in the appendix). The Discovery Studio (DS) 2020 software (BIOVIA Inc.: Shenzhen, Guangdong, China) used the three proteins mentioned above as receptor proteins. Furthermore, the structurally optimized S-THs molecules were used as ligand molecules in the LibDock module for rapid ligand-receptor docking to characterize the ligand-receptor binding ability using LDS. These include herbicidal functionality properties, degradability, and toxicity.

### 4.2. Characterization of the Comprehensive Effects of Herbicidal Functionality Properties, Microbial Degradability, and Human Lung Cytotoxicity of S-THs—AHP-TOPSIS Method

In this study, we used the Analytic Hierarchy Process—Technique for Order Preference by Similarity to the Ideal Solution (AHP-TOPSIS) [48] to normalize the herbicidal functionality properties, degradation, and toxicity indices of S-THs and calculated the comprehensive herbicidal functionality properties, degradation, toxicity, and CV of S-THs molecules according to the weighting ratios of the AHP-TOPSIS method. In addition, the CV was normalized using the AHP-TOPSIS method. The equations are as follows:

(1) Subjective weighting W1 (j) of herbicidal functionality properties, degradability, and toxicity of S-THs—SPSSAU software method

This study is primarily concerned with the herbicidal functionality properties of S-THs. The Analytic Hierarchy Process (AHP) module of SPSSAU software (QingSi Technology Ltd.: Beijing, China) was used to calculate the weight W1 (j) (the calculation and weighting methods are shown in the appendix).

(2) Objective weighting W_2_ (j) of herbicidal functionality properties, degradability, and toxicity of S-THs—TOPSIS weighting method

The docking scores of herbicidal functionality properties, degradability, and toxicity of S-THs were normalized in this study based on the type of indicator (positive and negative indicators, respectively), where herbicidal functionality properties and degradability are positive indicators, and toxicity is a negative indicator, and are calculated as follows:

Normalization of positive indicators of herbicidal functionality properties or degradability:

(1) $$Z_{ij}^{+}=\frac{X_{ij}-\min\{X_{ij}\}}{\max\left\{X_{ij}\right\}-\min\{X_{ij}\}}$$

Normalization of negative indicators of toxicity:

(2) $$Z_{ij}^{-}=\frac{\max\left\{X_{ij}\right\}-X_{ij}}{\max\left\{X_{ij}\right\}-\min\{X_{ij}\}}$$

where i denotes the S-THs molecule (i = 1, 2, …, 26), j denotes the receptor protein (j = 1, 2, 3 for 1FC9, 4L9X, and 6K1J, respectively), Z_ij_^+^ denotes the positive indicator of the S-THs molecule normalized for herbicidal functionality properties or degradability, Z_ij_^−^ denotes the negative indicator of the S-THs molecule normalized for toxicity, and X_ij_ denotes the molecular docking scoring value of the ith molecule to the jth protein.

The docking scores of herbicidal functionality properties, degradability, and toxicity of S-THs were used as column vectors a_1_, a_2_, and a_3_ to construct a 3 × 26 normalized data matrix (a_1_, a_2_, a_3_), and the best and worst values of each column were selected to construct the best vector A^+^ = (a_1_^+^, a_2_^+^, a_3_^+^) and the worst vector A^−^ = (a_1_^−^, a_2_^−^, a_3_^−^), respectively. The normalized data vector of each S-THs molecule was then compared to the best and worst vectors. Closer to the best vector indicates that the S-THs molecule’s comprehensive effect is better. In contrast, closer to the worst vector indicates that the S-THs molecule’s comprehensive effect is worse. Therefore, the elements of the optimal and worst vectors were calculated as follows:

(3) $$a_{j}^{+}=\left\{\begin{array}{c} \max\left(X_{ij}\right),X_{ij}\;is\;a\;\mathrm{positive}\;\mathrm{indicator}\\ \min\left(X_{ij}\right),X_{ij}\;is\;a\;\mathrm{negative}\;\mathrm{indicator} \end{array}\right.$$

(4) $$a_{j}^{-}=\left\{\begin{array}{c} \max\left(X_{ij}\right),X_{ij}\;is\;a\;\mathrm{positive}\;\mathrm{indicator}\\ \min\left(X_{ij}\right),X_{ij}\;is\;a\;\mathrm{negative}\;\mathrm{indicator} \end{array}\right.$$

where a_j_^+^ is the jth column element of the best vector and a_j_^−^ is the jth column element of the worst vector.

Based on the optimal and inferior vectors A^+^ and A^–^, the distances of each S-THs numerator from the optimal and inferior vectors were calculated, and a positive relative error matrix R^+^ = (r)_ij_^+^_3×26_ and a negative relative error matrix R^−^ = (r_ij_^−^)_3×26_ were constructed based on the ratio between them and the maximum distance.

(5) $$r_{ij}^{+}=\frac{\left|X_{ij}-a_{j}^{+}\right|}{\max\left(X_{ij}\right)-\min(X_{ij})}$$

(6) $$r_{ij}^{-}=\frac{\left|X_{ij}-a_{j}^{-}\right|}{\max\left(X_{ij}\right)-\min(X_{ij})}$$

where $r_{ij}^{+}$ denotes the element in row i, column j of the positive relative error matrix, and $r_{ij}^{-}$ denotes the element in row i, column j of the negative relative error matrix.

The cosine of the relative error angle between the herbicidal functionality properties, degradability, and toxicity indicators of the S-THs was calculated based on the relative error matrix θ_j_:

(7) $$\theta_{j}=cos<r_{ij}^{+},r_{ij}^{-}>=\frac{\sum_{i=1}^{m}r_{ij}^{+}\cdot r_{ij}^{-}}{\sqrt{\sum_{i=1}^{m}r_{ij}^{{+}^{2}}}\cdot \sqrt{\sum_{i=1}^{m}r_{ij}^{{-}^{2}}}}$$

The objective TOPSIS weights W_2_(j) for herbicidal functionality properties, degradability, and toxicity of the comprehensive effect of S-THs were calculated by normalizing the cosine of the relative error clincher.

(8) $$W_{2}(j)=\frac{\theta_{j}}{\sum_{j=1}^{m}\theta_{j}}$$

(3) The compound weighting of subjective and objective

The minF optimization problem was designed using the minimum entropy principle. The following are the calculated comprehensive weights w(j) of the herbicidal functionality properties, degradability, and toxicity of S-THs.

(9) $$minF=\sum_{j=1}^{m}w(j)\ln\frac{w(j)}{W_{1}(j)}+\sum_{j=1}^{m}w(j)\ln\frac{w(j)}{W_{2}(J)}$$

(10) $$s.t.\sum_{j=1}^{m}w(j)=1,w\left(j\right)>0$$

The comprehensive weights w (j) for herbicidal properties, degradability, and toxicity of the comprehensive effects of S-THs were calculated using the Lagrange multiplier method to solve the above equation. The subjective weights W_1_ (j) and objective weights W_2_ (j) was substituted to calculate the comprehensive weights.

(11) $$w\left(j\right)=\sqrt{W_{1}(j)W_{2}(j)}/\sum_{j=1}^{3}\sqrt{W_{1}(j)W_{2}(j)},\;j=1,2,3$$

### 4.3. Construction of a Model for the Comprehensive Effects of Herbicidal Functionality Properties, Microbial Degradability, and Human Lung Cytotoxicity of S-THs—3D-QSAR Model

The optimized molecules from the SYBYL-X2.0 software [49] Minimize module were classified into training and test sets in a 3:1 ratio randomly (template molecules were present at both the training and test levels; the molecular distribution of the model training and test sets is shown in Table S2), with the more widely used Propazine (PRZ) chosen as the template molecule. The Align Database module [50] was used for molecular stacking (the molecular structure and common backbone are shown in Figure S3).

The stacked training set molecules and docking scores were imported using the SYBYL-X2.0 software (where the comprehensive value was imported to construct the CoMSIA model [36,50] for the comprehensive effect of herbicidal properties, degradability and toxicity of S-THs). The Calculate Properties module output the calculated values for the hydrophobic (H), hydrogen bond acceptor (A), hydrogen bond donor (D), electrostatic (E), and steric fields (S) were output using the Calculate Properties module, and cross-verification and non-cross-verification [36,50]. The molecules from the superimposed test set were then imported into SYBYL-X2.0 software. Based on the analysis results of the CoMSIA model constructed using the training set molecules, the predicted value of the test set molecule output was obtained using the Predict function under the Add a Computed Column module [36,50]. Finally, using the Calculate Properties module [36,50], the predicted values were externally validated against the original scoring values (the comprehensive model for the comprehensive values). After passing all the above validations, the built model of the herbicidal, degradation, and toxicity effects of S-THs and their comprehensive effects proved stable, predictive, and well-fitting. The parameters and model evaluation criteria for the training sets and test sets of the CoMSIA model developed in this study are listed in Table S11 [36].

### 4.4. Design of S-TH Substitutes—SYBYL-X2.0 Software

In this study, using the SYBYL-X2.0 software, we propose the selection of the most widely used ATZ as a template molecule and determine the substitutable group sites and substitution groups based on the 3D isopotential diagrams of each force field (including hydrophobic field (H), hydrogen bond acceptor field (A), hydrogen bond donor field (D), electrostatic field (E), and steric field (S)) of the constructed CoMSIA model of the comprehensive effect of herbicidal properties, degradability, and toxicity of S-THs. The introduction of hydrophobic substituents (-CH_3_, CF_3_, -F/Cl/Br, -OCH_3_, and -SH) near the yellow region of the hydrophobic field (H) and hydrophilic substituents (-OH, -CHO, -COOH, and -NH_2_) near the white region of the hydrophobic field (A) improved the activities of the compounds. The addition of hydrogen bond acceptors (-NO_2_, NF_2_, and -COCF_3_) in the purple region of the hydrogen bond acceptor field (A) and hydrogen bond donors (-NH_2_, -OH, -COCH_3_, and -CONH_2_) in the red region of the field improved the activities of the compounds. The addition of hydrogen bond donors in the cyan region of the hydrogen bond donor field (D) and hydrogen bond acceptors in the purple region of the field improved the activities of the compounds. Introducing less electronegative substituents (-H, -OH, -COOH, and –NH) in the blue region of the electrostatic field (E) improved the activities of the compounds. The introduction of less electronegative substituents (-H, -OH, and -CH_3_) in the blue region of the electrostatic field (E) and more electronegative substituents (-CF_3_, -F, -CH_2_ F, and -CHF_2_) in the red region of the field improved the activities of the compounds. The activities of the compounds were improved by increasing the size of the substituents in the green region of the steric field (S) and decreasing the size in the yellow region [51]. The Sketch Molecule, Minimize, and Align Database modules of the SYBYL-X2.0 software were used to map and optimize substitutes [36].

### 4.5. Evaluation of the Microbial Degradability Universality and Toxicity of Antioxidant Systems in Fish of S-THs Substitutes—Molecular Docking Method

#### 4.5.1. Evaluation of the Microbial Degradability Universality of S-THs Substitutes in the Soil Environment

**Figure 5:**
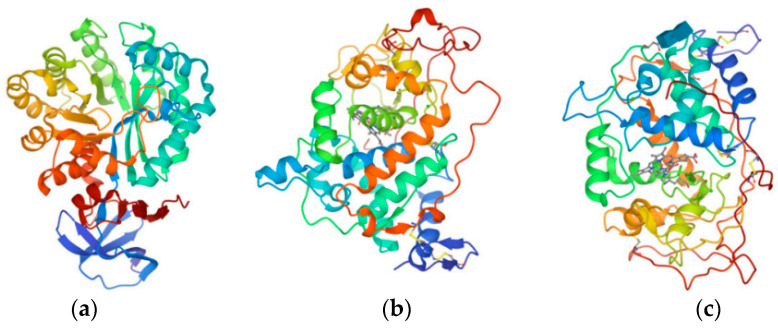
Schematic diagram of the structure of a universal degrading protein of S-TH substitutes in the soil environment: (**a**) 2QT3; (**b**) 1B85; (**c**) 1MNP.

The PDB database was used to search and select three proteins as the target proteins of microbial degradation (Figure 5, the rationale for the selection of the above three proteins is shown in the appendix), which are AtzC of *Pseudomonas* sp. Migula (Pseudomonadales: Pseudomonadaceae) ADP (PDB ID: 2QT3), LiP of *Phanerodontia chrysosporium* Burds. (Polyporales: Phanerochaetaceae) (PDB ID: 1B85), and MnP (PDB ID: 1MNP). The LibDock module of DS software was used to perform molecular docking between the designed S-TH substitutes and the above proteins. LDS was used to characterize the microbial degradability of S-TH substitutes, which was used as an evaluation index of the microbial degradability universality of S-TH substitutes in the the soil environment.

#### 4.5.2. Evaluation of the Toxicity of Antioxidant Systems in Fish of S-TH Substitutes in the Aquatic Environment

**Figure 6:**
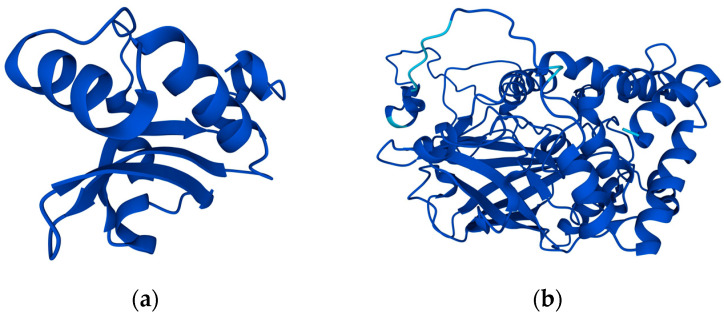
Schematic representation of antioxidant proteins in carp: (**a**) Q8JFG7; (**b**) E2CWE8.

The UniProt database [34] was used to identify superoxide dismutase (SOD, UniProt ID: Q8JFG7), and hydrogen peroxide proteins (CAT, UniProt ID: E2CWE8) (Figure 6) as receptor proteins in carp (the rationale for the selection of the above three proteins is shown in the appendix). The above proteins were molecularly docked with S-TH substitutes with universal microbial degradability using the LibDock module of the DS software. Protein activity was more likely to be inhibited when the docking score was higher. The docking scores of the two proteins were added at a weight of 1:1, and the comprehensive value was used to characterize the toxic effect of S-TH substitutes on the antioxidant system of fish. The higher the comprehensive value, the stronger the toxicity of S-TH substitutes on the antioxidant system of fish.

### 4.6. Screen of Optimal Field Application Schemes to Promote the Microbial Degradation of S-TH Substitutes in Maize Cropping Fields—Taguchi Orthogonal Experiments, Full Factorial Design of Experiments, and Molecular Dynamics Methods

THs are applied primarily to maize [1]. In this study, the maize cropping field was selected as the primary research area to explore the influence of different external stimulus conditions (such as fertilizer and crop root secretion) of the maize cropping field on the microbial degradability of S-TH substitutes in the soil environment and to screen and determine the best field application scheme of S-TH substitutes. Furthermore, root exudates can promote microbial degradation near the rhizosphere [40]. In this study, 18 compounds, including the most widely used nitrogen fertilizer [52] and 17 kinds of rhizosphere secretory substances of maize, were selected (Table S12) [53,54] as external addictive conditions to investigate their influence on the degradability of S-TH substitutes.

In this study, 18 compounds (Table S12) were chosen as external conditions to design an 18-factors and 2-levels (0 for no addition, 1 for addition) Taguchi orthogonal experiment, with a total of 32 different sets of external conditions schemes. In addition, the binding energy of the substitute D-5, triazine hydrolysate protein 4L9X, and the complex system of external conditions (Table S7) were calculated to screen the optimal field application schemes to promote the microbial degradation of S-TH substitutes preliminary.

The Taguchi orthogonal experimental design is a method for independently evaluating the single-factor-level effects [55]. Eighteen compounds were chosen as external additives in this study. The Taguchi (T) module under the design of the experiment module in Minitab 20 software was used to construct a Taguchi orthogonal experimental design with 18 factors and two levels (0 represents no addition, 1 represents addition). A total of 32 groups of external condition-adding schemes were included. Among them, the S-TH substitutes and 4L9X proteins selected as fixed conditions were added to this scheme’s molecular dynamics calculation system.

The Gromacs 4.6.5 software (GROMACS development team: Stockholm, Sweden) [56] was used to simulate the molecular dynamics of the 32 groups of schemes. After docking, the compound system of S-TH substitutes, external condition compounds, and 4L9X protein were placed in a periodic cubic aqueous solution with a side length of 15 nm. The GROMOS96 43a1 force field was utilized for molecular restraint. In addition, a positively charged Na^+^ neutralizing system was added. The binding energy between S-TH substitutes, external condition compounds, and 4L9X proteins in each group was calculated using the Molecular Mechanics/Poisson-Boltzmann Surface Area (MMPBSA) method (the smaller the binding energy, the stronger the promoting effect of the scheme on the microbial degradation of S-TH substitutes). Finally, the external condition addition scheme with the minimum binding energy was used as the initial screening scheme for the subsequent total factorial experimental design. Furthermore, the best field application scheme is conducive to degrading S-TH substitutes by soil rhizosphere microorganisms in maize cropping fields.

The full factorial design of experiments is a method that allows rapid screening of multilevel, multifactorial, and correlated vital factors, thereby reducing experimental workload and increasing efficiency [57]. In this study, we used the Factor (F) module under the design of the experiment module of the Minitab 20 software (Minitab LLC.: Centre County, Pennsylvania, United States) to construct an n-factor 2-level full factorial design of the experiment based on the initial screening scheme of the Taguchi orthogonal experiment (assuming the number of external conditions in the scheme is n). Molecular dynamics was used to calculate the binding energies of S-THs, external conditions, and 4L9X protein. In addition, using Minitab 20 software, the absolute values of binding energies were entered as response values into the constructed design of the experimental table, and the factorial design was analyzed using the Factorial (F) module under the design of the experimental module. The interaction mechanisms (synergistic and antagonistic) between the n-factors were further analyzed to verify the reliability of the screened optimal field application options.

### 4.7. Simulation of Microbial Degradation Pathways of S-TH Substitutes—DFT and Microbial Degradation Pathway Simulation

In this study, the microbial degradation pathways of S-THs before and after molecular modification were simulated and inferred from that of ATZ indicated in the literature (Figure S4) [58]. Furthermore, the possible intermediate and final products produced during the degradation and the change of microbial degradation degree molecules before and after modification were analyzed based on DFT. Gaussian09 software (Gaussian Inc. Wallingford, Connecticut, United States) was used to optimize and calculate the reaction energy barrier (ΔE > 0, with a smaller energy barrier indicating a more accessible reaction) of the microbial degradation of S-THs before and after molecular modification at the B3LYP/6-31G* unit level [35,59]. It has been shown that the ΔE (>0) represents the difficulty of reaction occurrence, with smaller values indicating that the reaction is more likely to occur [35]. Therefore, the ΔE results were used to evaluate the microbial degradation of S-THs before and after molecular modification.

### 4.8. Evaluation of Aquatic Biotoxicity and Human Health Risks of Microbial Degradation Products of S-TH Substitutes—EPI Software Method, Pharmacokinetic and Toxicokinetic Methods

Estimation Programs Interface (EPIWEB 4.1) software (SRC Inc.: Syracuse, New York, NY, USA) [60] was used in this study to predict and evaluate the aquatic toxicity of intermediate microbial degradation products by calculating the toxicity of the aquatic organisms (green algae (EC_50_), Daphnia (LC_50_), and fish (LC_50_)). In addition, the pharmacokinetic and toxicokinetic methods in the ADMET module of the DS software were used to calculate the human health risks (hepatotoxicity, maximum tolerated dosage, skin sensitization, skin irritation, and rodent carcinogenicity) of S-THs and their substitutes’ microbial degradation products. These indicators can be used to predict and evaluate the human health risks posed by microbial degradation products.

## 5. Conclusions

This study developed an ecological and sustainable S-THs control scheme that can effectively reduce the environmental and human health impacts of S-THs application in maize cropping fields through molecular source prevention, field application process control, and end-of-soil degradation evaluation. The main findings were as follows: (1) the design of a substitute to S-THs with high herbicidal functionality and microbial degradability, low human health risk, and environmental friendliness using three-dimensional quantitative structure-activity relationship (3D-QSAR) modeling and molecular docking methods; (2) the simulation and screening of fertilizer and soil secretion that can promote microbial degradation of the substitute to S-THs in maize cropping fields using Taguchi orthogonal experiments and full factorial design of experiments; and (3) Based on the DFT, we simulated and calculated the degradation pathways and reaction energy barrier of S-TH molecules before and after modification, and confirmed that the designed S-TH substitutes have stronger microbial degradability. This study developed a source modification scheme for S-TH substitutes, an optimal application process control scheme for S-TH substitutes in maize cropping fields, and an environmental and human health evaluation scheme for their terminal potential degradation products, which provides theoretical guidance for minimizing the risk of S-THs application to the environment and human health in maize cropping fields.

## Figures and Tables

**Table 1:** CoMSIA model evaluation parameters for the herbicidal functionality properties, degradability, and toxicity of S-THs and their comprehensive effects.

CoMSIA Models	Enzymes	q^2^	n	R^2^	SEE	F	r^2^_pred_	(R^2^ − q^2^)/R^2^ (%)
Comprehensive	ALL	0.789	8	0.993	0.007	132.292	0.615	20.54
Herbicide	1FC9	0.751	9	0.997	0.393	207.333	0.785	24.67
Degradation	4L9X	0.757	5	0.986	1.529	157.196	0.713	23.23
Toxicity	6K1J	0.706	10	1.000	0.285	1660.695	0.792	29.40

**Table 2:** The proportion of each force field in the single-effect and comprehensive-effect CoMSIA models of herbicidal functionality properties, degradability, and toxicity of S-THs based on the 3D isopotential diagrams.

Fields	Proportion of Fields (%)			
	CoMSIA Models			
	For Comprehensive Activity	For Herbicidal Activity	For Degradation Activity	For Toxicity Activity
Hydrophobic (H)	37.4	50	29.3	32.5
Hydrogen-bond acceptor (A)	4.7	6.9	7.1	6.9
Hydrogen-bond donor (D)	26.2	6.8	27.6	22.8
Electrostatic (E)	16.8	18.8	17.8	19.4
Steric (S)	14.9	16.5	18.1	18.4

**Table 3:** Calculation of reaction energy barrier and change rates of the microbial degradation of ATZ and substitute D-5 in Stage 1.

Compounds	Steps	Reactants	Products	ΔE	ΔE (Total)	Change Rate (%)
				(kJ/mol)	(kJ/mol)	
ATZ	1	ATZ	ATZ-1	160.893	200.806	-
	2	ATZ-1	Com-1	39.913		
D-5	1	D-5	D-5-1	60.147	94.533	−62.62
	2	D-5-1	Com-1	34.386		−13.85

## Data Availability

Data is contained within the article or supplementary material.

## Supplementary Materials

The following supporting information can be downloaded at: https://www.mdpi.com/article/10.3390/ijms24065691/s1, Figure S1: The outcomes of a comprehensive weighting of herbicidal, degradability, and toxicity for the comprehensive effect of S-TH molecules; Figure S2: Molecular structure of ATZ with proposed modification sites (Site 1: -CH_3_; Site 2: -H; Site 3: -H; Site 4: -CH_3_, -CH_3_; Site 5: -Cl); Figure S3: The structure and the common backbone of PRZ; Figure S4: Microbial degradation pathway of ATZ; Table S1: Docking scores for herbicidal, degradative, and toxic effects of S-TH molecules and calculation of their comprehensive value; Table S2: Molecular combination of the CoMSIA model training sets and test sets; Table S3: Substitution sites and substituent groups for the design of molecular substitutes to S-THs; Table S4: Predicted values of herbicidal, degradative, and toxic effects of molecular substitutes to S-THs, their comprehensive effects, and rates of change; Table S5: The LDS values and their rates of change for the microbial degradation pervasiveness of molecular substitutes to S-THs; Table S6: Comprehensive values of the toxic effects of molecular substitutes to S-THs on the antioxidant system of fish; Table S7: Molecular dynamics simulations of the external condition addition scheme (18 factors/2 levels) based on the Taguchi orthogonal experiments; Table S8: Response and effect values for the external condition addition scenario (3 factors/2 levels); Table S9: Evaluation of the aquatic toxicity of D-5 microbial degradation products based on the EPI software method; Table S10: Human health risk assessment of D-5 microbial degradation products based on pharmacokinetic and toxicokinetic data; Table S11: CoMSIA model evaluation methods and model evaluation criteria; Table S12: Nitrogen fertilizer and maize root secretion and their corresponding factors.
