# Supplementary material for: Triazine Herbicides Risk Management Strategies on Environmental and Human Health Aspects Using In-Silico Methods
Source: Int J Mol Sci. 2023 Mar 16;24(6):5691. doi: 10.3390/ijms24065691 (PMC10052965; doi:10.3390/ijms24065691)
Supplement: Supplementary file 1 [file ijms-24-05691-s001.zip › ijms-2257972-supplementary.pdf]

Supporting information

Triazine Herbicides Risk Management Strategies on Environmental and Human Health Aspects Using In-Silico Methods

Tianfu Yao †, Peixuan Sun † and Wenjin Zhao *

College of New Energy and Environment, Jilin University, Changchun 130012, China

* Correspondence: zhaowj@jlu.edu.cn

† These authors contributed equally to this work.

2. Results

2.1 Construction and evaluation of the single-effect and comprehensive-effect 3D-QSAR models of herbicidal functionality properties, microbial degradability, and human lung cytotoxicity of S-THs

Table S1. Docking scores for herbicidal, degradative, and toxic effects of S-TH molecules and calculation of their comprehensive value

No.	Compounds	LibDock Score (LDS)			Comprehensive value
		1FC9	4L9X	6K1J	(CV)
1	Propazine	0.385	0.433	0.556	0.440
2	Atrazine	0.349	0.438	0.590	0.433
3	Simazine	0.331	0.415	0.638	0.431
4	Sebuthylazine	0.419	0.491	0.484	0.454
5	Sebuthylazine-desethyl	0.333	0.306	0.597	0.394
6	Terbuthylazine	0.190	0.489	0.574	0.363
7	Cyprazine	0.399	0.485	0.486	0.443
8	Trietazine	0.345	0.414	0.566	0.419
9	Desmetryn	0.192	0.384	0.602	0.345
10	Simetryn	0.195	0.413	0.593	0.351
11	Methoprotryne	0.687	0.662	0.309	0.584
12	Dimethametryn	0.259	0.627	0.420	0.393
13	Prometryn	0.292	0.504	0.515	0.402
14	Terbutryn	0.226	0.497	0.469	0.356
15	Dipropetryn	0.374	0.565	0.455	0.443
16	Simeton	0.362	0.414	0.534	0.419
17	Atraton	0.351	0.511	0.530	0.437
18	Prometon	0.391	0.493	0.520	0.450
19	Secbumeton	0.396	0.572	0.429	0.449
20	Terbumeton	0.209	0.520	0.443	0.347
21	Cyanazine	0.347	0.495	0.427	0.405

22	Deethylatrazine	0.236	0.211	0.681	0.343
23	Deethyldeisopropylatrazine	0.000	0.000	1.000	0.255
24	Triaziflam	1.000	1.000	0.000	0.745
25	Indaziflam	0.279	0.883	0.159	0.400
26	Ametryn	0.226	0.490	0.529	0.370

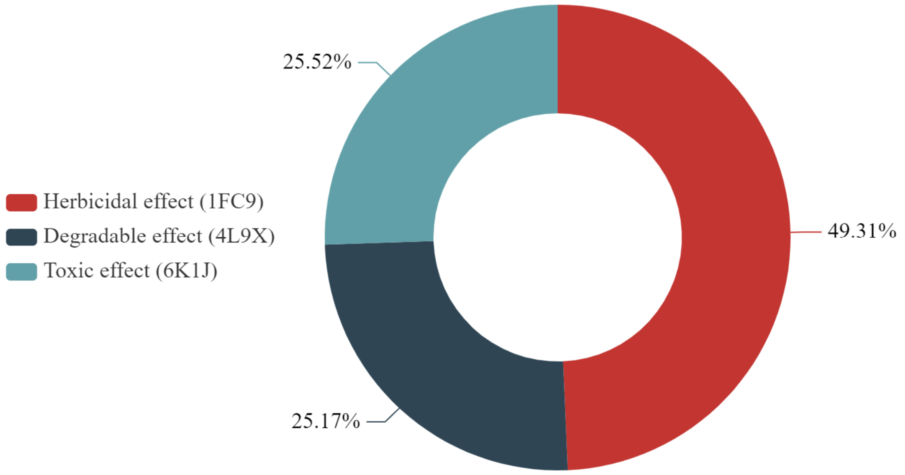

Figure S1. The outcomes of a comprehensive weighting of herbicidal, degradability, and toxicity for the comprehensive effect of S-TH molecules

Table S2. Molecular combination of the CoMSIA model training sets and test sets

**Molecules**	**Comprehensive activity**		**Herbicidal activity**		**Degradation activity**		**Toxicity activity**	
	**Training**	**Text**	**Training**	**Text**	**Training**	**Text**	**Training**	**Text**
Propazine	Template Molecule							
Atrazine	√		√		√		√	
Simazine	√					○	√	
Sebuthylazine	√			○	√		√	
Sebuthylazine-desethyl	√					○	√	
Terbuthylazine	√		√					○
Cyprazine	√					○	√	
Ttrietazine			√					
Desmetryn				○		○	√	
Simetryn	√		√		√			○

Methoprotryne					√		√	
Dimethametryn	√			○		○		○
Prometryn		○	√			○		○
Terbutryn		○	√		√		√	
Dipropetryn	√		√		√		√	
Simeton		○	√		√			○
Atraton	√		√		√		√	
Prometon		○	√		√			
Secbumeton	√		√		√		√	
Terbumeton	√		√		√		√	
Cyanazine		○	√		√		√	
Deethylatrazine	√			○	√			
Deethyldeisopropylatrazine	√			○	√		√	
Triaziflam								
Indaziflam			√					
Ametryn	√		√		√		√	

2.2 Design of S-THs substitutes based on the 3D isopotential diagrams of CoMSIA model

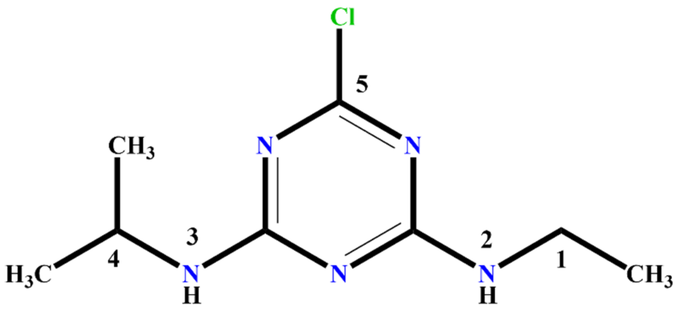

Figure S2. Molecular structure of ATZ with proposed modification sites (Site 1: -CH_3; Site 2: -H; Site 3: -H; Site 4: -CH_3, -CH_3; Site 5: -Cl)

Table S3. Substitution sites and substituent groups for the design of molecular substitutes to S-THs

No.	Compounds	Substituent sites and groups
D-1	Derivative-1	1-Fluorine, 1-Fluorine, 3-Trifluoromethyl, 4-Methyl
D-2	Derivative-2	1-Fluorine, 1-Fluorine, 1-Bromine, 3-Trifluoromethyl, 4-Methyl

D-3	Derivative-3	1-Fluorine, 1-Fluorine, 1-Ethynyl, 3-Trifluoromethyl, 4-Methyl
D-4	Derivative-4	1-Fluorine, 1-Fluorine, 1-Trifluoromethyl, 3-Trifluoromethyl, 4-Methyl
D-5	Derivative-5	1^S-Fluorine, 1^S-Mercapto, 1-Trifluoromethyl, 3-Trifluoromethyl, 4-Methyl
D-6	Derivative-6	1^S-Fluorine, 1^S-Chlorine, 1^S-Ethynyl, 3-Trifluoromethyl, 4-Methyl
D-7	Derivative-7	1^S-Fluorine, 1^S-Bromine, 1^S-Ethynyl, 3-Trifluoromethyl, 4-Methyl
D-8	Derivative-8	1-Chlorine, 1-Chlorine, 1-Ethynyl, 3-Trifluoromethyl, 4-Methyl
D-9	Derivative-9	1^R-Fluorine, 1^R-Chlorine, 1^R-Ethynyl, 3-Trifluoromethyl, 4-Methyl
D-10	Derivative-10	1^S-Bromine, 1^S-Chlorine, 1^S-Ethynyl, 3-Trifluoromethyl, 4-Methyl
D-11	Derivative-11	1-Bromine, 1-Bromine, 1-Ethynyl, 3-Trifluoromethyl, 4-Methyl
D-12	Derivative-12	1^S-Fluorine, 1^S-Mercapto, 1^S-Ethynyl, 3-Trifluoromethyl, 4-Methyl
D-13	Derivative-13	1^S-Chlorine, 1^S-Mercapto, 1^S-Ethynyl, 3-Trifluoromethyl, 4-Methyl
D-14	Derivative-14	1^S-Bromine, 1^S-Mercapto, 1^S-Ethynyl, 3-Trifluoromethyl, 4-Methyl
D-15	Derivative-15	1^R-Fluorine, 1^R-Mercapto, 1^R-Ethynyl, 3-Trifluoromethyl, 4-Methyl
D-16	Derivative-16	1-Fluorine, 1-Fluorine, 1-Monofluoromethyl, 3-Trifluoromethyl, 4-Methyl
D-17	Derivative-17	1-Fluorine, 1-Fluorine, 1-Ethenyl, 3-Trifluoromethyl, 4-Methyl
D-18	Derivative-18	1-Fluorine, 1-Fluorine, 1-Difluoromethyl, 3-Trifluoromethyl, 4-Methyl
D-19	Derivative-19	1-Fluorine, 1-Fluorine, 1-Aldehyde, 3-Trifluoromethyl, 4-Methyl
D-20	Derivative-20	1-Fluorine, 1-Fluorine, 1-Chlorine, 3-Trifluoromethyl, 4-Methyl
D-21	Derivative-21	1-Fluorine, 1-Fluorine, 1-Cyano, 3-Trifluoromethyl, 4-Methyl
D-22	Derivative-22	1-Fluorine, 1-Fluorine, 1-Acetyl, 3-Trifluoromethyl, 4-Methyl
D-23	Derivative-23	1-Fluorine, 1-Fluorine, 1-Aminoacetyl3-Trifluoromethyl, , 4-Methyl
D-24	Derivative-24	1-Fluorine, 1-Fluorine, 1-Carboxyl, 3-Trifluoromethyl, 4-Methyl
D-25	Derivative-25	1-Fluorine, 1-Fluorine, 1-Fluorine, 3-Trifluoromethyl, 4-Methyl
D-26	Derivative-26	1-Fluorine, 1-Fluorine, 3-Trifluoromethyl, 4-Methyl
D-27	Derivative-27	1^S-Fluorine, 1^S-Mercapto, 1^S-Ethynyl, 3-Trifluoromethyl, 4-Methyl
D-28	Derivative-28	1-Fluorine, 1-Fluorine, 1-Nitro, 3-Trifluoromethyl, 4-Methyl
D-29	Derivative-29	1-Fluorine, 1-Fluorine, 1-Methoxy, 3-Trifluoromethyl, 4-Methyl
D-30	Derivative-30	1^R-Fluorine, 1^R-Chlorine, 1-Methoxy, 3-Trifluoromethyl, 4-Methyl
D-31	Derivative-31	1^S-Fluorine, 1^S-Chlorine, 1-Methoxy, 3-Trifluoromethyl, 4-Methyl

D-32	Derivative-32	1-Fluorine, 1-Fluorine, 1-Hydroxyl, 3-Trifluoromethyl, 4-Methyl
D-33	Derivative-33	1-Fluorine, 1-Fluorine, 1-Mercapto, 3-Trifluoromethyl, 4-Methyl
D-34	Derivative-34	1-Fluorine, 1-Fluorine, 1-Amino, 3-Trifluoromethyl, 4-Methyl
D-35	Derivative-35	1^R-Fluorine, 1^R-Trifluoromethyl, 3-Trifluoromethyl, 4-Methyl
D-36	Derivative-36	1^R-Fluorine, 1^R-Monofluoromethyl, 3-Trifluoromethyl, 4-Methyl
D-37	Derivative-37	1^R-Fluorine, 1^R-Difluoromethyl, 3-Trifluoromethyl, 4-Methyl
D-38	Derivative-38	1^R-Fluorine, 1^R-Methoxy, 3-Trifluoromethyl, 4-Methyl
D-39	Derivative-39	1^S-Fluorine, 1^S-Trifluoromethyl, 3-Trifluoromethyl, 4-Methyl
D-40	Derivative-40	1^S-Fluorine, 1^S-Methoxy, 3-Trifluoromethyl, 4-Methyl

2.3 Prediction and evaluation of the single-effect and comprehensive-effect 3D-QSAR models of herbicidal properties, microbial degradability, and human lung cytotoxicity of S-THs

Table S4. Predicted values of herbicidal, degradative, and toxic effects of molecular substitutes to S-THs, their comprehensive effects, and rates of change

Molecules	CV		Herbicidal effect		Degradable effect		Toxic effect	
			1FC9		4L9X		6K1J	
	Pred.	Change rate (%)	Pred.	Change rate (%)	Pred.	Change rate (%)	Pred.	Change rate (%)
ATZ	0.433		0.263		0.312		0.228	
D-1	0.615	42.03	0.672	155.58	0.795	154.66	0.309	35.34
D-2	0.641	48.04	0.754	186.55	0.525	68.36	0.639	180.43
D-3	0.631	45.73	0.713	171.19	0.681	118.27	0.449	96.72
D-4	0.700	61.66	0.872	231.66	0.674	116.15	0.494	116.81
D-5	0.697	60.97	0.879	234.39	0.668	114.04	0.472	107.02
D-6	0.64	47.81	0.752	186.12	0.754	141.53	0.370	62.25
D-7	0.632	45.96	0.734	178.96	0.774	148.18	0.340	49.21
D-8	0.656	51.50	0.781	196.83	0.797	155.34	0.316	38.60
D-9	0.647	49.42	0.742	181.98	0.729	133.79	0.388	70.09
D-10	0.649	49.88	0.763	189.99	0.814	160.90	0.291	27.47
D-11	0.642	48.27	0.749	184.71	0.826	164.71	0.269	17.93

D-12	0.633	46.19	0.720	173.79	0.680	117.81	0.421	84.55
D-13	0.657	51.73	0.804	205.59	0.772	147.47	0.330	44.79
D-14	0.643	48.50	0.762	189.71	0.775	148.43	0.313	37.48
D-15	0.626	44.57	0.737	180.26	0.703	125.47	0.403	76.85
D-16	0.591	36.49	0.633	140.85	0.692	121.82	0.431	89.13
D-17	0.627	44.80	0.696	164.75	1.000	220.51	0.061	-73.20
D-18	0.636	46.88	0.710	170.04	0.672	115.48	0.456	100.05
D-19	0.593	36.95	0.629	139.33	0.519	66.49	0.606	165.96
D-20	0.645	48.96	0.761	189.32	0.501	60.64	0.670	193.81
D-21	0.645	48.96	0.758	188.17	0.476	52.53	0.711	211.84
D-22	0.694	60.28	0.854	224.64	0.740	137.14	0.389	70.69
D-23	0.567	30.95	0.621	136.18	0.341	9.40	0.816	257.88
D-24	0.613	41.57	0.718	173.10	0.453	45.33	0.706	209.55
D-25	0.653	50.81	0.778	195.78	0.450	44.26	0.734	221.77
D-26	0.608	40.42	0.684	160.12	0.583	86.86	0.566	148.08
D-27	0.592	36.72	0.678	157.97	0.355	13.79	0.836	266.58
D-28	0.317	-26.79	0.000	-100.00	0.090	-71.09	1.000	338.60
D-29	0.645	48.96	0.743	182.43	0.625	100.39	0.513	124.81
D-30	0.642	48.27	0.737	180.05	0.671	114.92	0.450	97.32
D-31	0.658	51.96	0.761	189.54	0.647	107.38	0.479	109.89
D-32	0.633	46.19	0.746	183.68	0.432	38.39	0.750	229.06
D-33	0.738	70.44	1.000	280.23	0.607	94.43	0.604	164.95
D-34	0.501	15.70	0.447	70.13	0.000	-100.00	0.489	114.69
D-35	0.637	47.11	0.771	193.25	0.642	105.63	0.484	112.08
D-36	0.537	24.02	0.484	84.03	0.659	111.34	0.400	75.43
D-37	0.544	25.64	0.489	85.82	0.734	135.18	0.307	34.69
D-38	0.595	37.41	0.593	125.55	0.713	128.43	0.352	54.42
D-39	0.511	18.01	0.442	67.93	0.499	59.80	0.000	-100.00
D-40	0.617	42.49	0.672	155.47	0.671	115.06	0.406	78.18

2.4 Evaluation of the microbial degradability universality and toxicity of antioxidant systems in fish of S-THs substitutes

2.4.1 Evaluation of the microbial degradability universality of S-THs substitutes

Table S5. The LDS values and their rates of change for the microbial degradation pervasiveness of molecular substitutes to S-THs

No.	LDS					
	2QT3	Change rate (%)	1B85	Change rate (%)	1MNP	Change rate (%)
ATZ	85.451		95.133		88.089	
D-3	100.219	17.28	98.258	3.29	102.273	16.10
D-4	97.921	14.59	98.302	3.33	95.286	8.17
D-5	97.373	13.95	93.455	-1.76	97.985	11.23
D-18	90.499	5.91	102.915	8.18	97.624	10.82
D-29	94.812	10.95	97.925	2.94	95.993	8.97
D-30	93.245	9.12	97.465	2.45	101.225	14.91
D-31	93.096	8.95	96.013	0.92	93.803	6.49
D-35	100.085	17.13	101.125	6.30	108.872	23.59

2.4.2 Evaluation of the toxicity of antioxidant systems in fish of S-THs substitutes

Table S6. Comprehensive values of the toxic effects of molecular substitutes to S-THs on the antioxidant system of fish

No.	LDS		CV	Change rate (%)
	SOD	CAT		
Atrazine	71.031	94.491	82.761	-
D-3	73.733	103.707	88.720	7.20
D-4	75.214	83.483	79.349	-4.12
D-5	44.262	81.563	62.912	-23.98
D-18	74.459	107.362	90.911	9.85
D-29	57.617	99.243	78.430	-5.23
D-30	70.286	104.348	87.317	5.51

D-31	66.884	98.437	82.661	-0.12
D-35	46.408	97.46	71.934	-13.08

2.5 Screen of optimal field application schemes to promote the microbial degradation of S-THs substitutes in maize cropping fields

Table S7. Molecular dynamics simulations of the external condition addition scheme (18 factors / 2 levels) based on the Taguchi orthogonal experiments

No.	Factors																		Binding energy (kJ/mol)	Change rate (%)
	A	B	C	D	E	F	G	H	J	K	L	M	N	O	P	Q	R	S		
1	0	0	0	0	0	0	0	0	0	0	0	0	0	0	0	0	0	0	-93.414	-
2	0	0	0	0	0	0	0	0	0	0	0	0	0	0	0	1	1	1	-147.893	-58.32
3	0	0	0	0	0	0	0	1	1	1	1	1	1	1	1	0	0	0	-91.423	2.13
4	0	0	0	0	0	0	0	1	1	1	1	1	1	1	1	1	1	1	-59.048	36.79
5	0	0	0	1	1	1	1	0	0	0	0	1	1	1	1	0	0	0	-88.737	5.01
6	0	0	0	1	1	1	1	0	0	0	0	1	1	1	1	1	1	1	-48.786	47.77
7	0	0	0	1	1	1	1	1	1	1	1	0	0	0	0	0	0	0	-75.952	18.69
8	0	0	0	1	1	1	1	1	1	1	1	0	0	0	0	1	1	1	-40.316	56.84
9	0	1	1	0	0	1	1	0	0	1	1	0	0	1	1	0	0	1	-88.267	5.51
10	0	1	1	0	0	1	1	0	0	1	1	0	0	1	1	1	1	0	-103.369	-10.66
11	0	1	1	0	0	1	1	1	1	0	0	1	1	0	0	0	0	1	-81.848	12.38
12	0	1	1	0	0	1	1	1	1	0	0	1	1	0	0	1	1	0	-73.892	20.90
13	0	1	1	1	1	0	0	0	0	1	1	1	1	0	0	0	0	1	-86.145	7.78
14	0	1	1	1	1	0	0	0	0	1	1	1	1	0	0	1	1	0	-111.816	-19.70
15	0	1	1	1	1	0	0	1	1	0	0	0	0	1	1	0	0	1	-96.277	-3.06
16	0	1	1	1	1	0	0	1	1	0	0	0	0	1	1	1	1	0	-85.199	8.79
17	1	0	1	0	1	0	1	0	1	0	1	0	1	0	1	0	1	0	-80.375	13.96
18	1	0	1	0	1	0	1	0	1	0	1	0	1	0	1	1	0	1	-61.983	33.65
19	1	0	1	0	1	0	1	1	0	1	0	1	0	1	0	0	1	0	-103.571	-10.87
20	1	0	1	0	1	0	1	1	0	1	0	1	0	1	0	1	0	1	-78.853	15.59

21	1	0	1	1	0	1	0	0	1	0	1	1	0	1	0	0	1	0	-94.825	-1.51
22	1	0	1	1	0	1	0	0	1	0	1	1	0	1	0	1	0	1	-79.883	14.48
23	1	0	1	1	0	1	0	1	0	1	0	0	1	0	1	0	1	0	-84.956	9.05
24	1	0	1	1	0	1	0	1	0	1	0	0	1	0	1	1	0	1	-83.301	10.83
25	1	1	0	0	1	1	0	0	1	1	0	0	1	1	0	0	1	1	-78.682	15.77
26	1	1	0	0	1	1	0	0	1	1	0	0	1	1	0	1	0	0	-102.712	-9.95
27	1	1	0	0	1	1	0	1	0	0	1	1	0	0	1	0	1	1	-98.862	-5.83
28	1	1	0	0	1	1	0	1	0	0	1	1	0	0	1	1	0	0	-103.880	-11.20
29	1	1	0	1	0	0	1	0	1	1	0	1	0	0	1	0	1	1	-105.482	-12.92
30	1	1	0	1	0	0	1	0	1	1	0	1	0	0	1	1	0	0	-87.455	6.38
31	1	1	0	1	0	0	1	1	0	0	1	0	1	1	0	0	1	1	-85.413	8.57
32	1	1	0	1	0	0	1	1	0	0	1	0	1	1	0	1	0	0	-81.190	13.09

Note: “0” means not added, “1” means added

Table S8. Response and effect values for the external condition addition scenario (3 factors / 2 levels)

No.	Factors			Response	Change rate	Effect
	Q	R	S	values	(%)	values
1	0	0	0	93.414	-	-
2	1	0	0	95.096	1.80	20.77
3	0	1	0	117.223	25.49	13.706
4	1	1	0	120.732	29.24	15.112
5	0	0	1	106.259	13.75	8.304
6	1	0	1	120.892	29.42	20.67
7	0	1	1	79.639	-14.75	-13.516
8	1	1	1	147.893	58.32	11.699

2.7 Evaluation of aquatic biotoxicity and human health risks of microbial degradation products of S-THs substitutes

Table S9. Evaluation of the aquatic toxicity of D-5 microbial degradation products based on the EPI software method

No.	Green algae EC_{50}	Change rate (%)	DAP LC_{50}	Change rate (%)	Fish LC_{50}	Change rate (%)
ATZ	0.079	-	17.631	-	21.731	-
D-5-P1	21.648	27402.53	19.664	11.53	190.844	778.21
D-5-P2	16.744	21094.94	15.167	-13.98	147.411	578.34

Table S10. Human health risk assessment of D-5 microbial degradation products based on pharmacokinetic and toxicokinetic data

No.	Hepatotoxicity		Rat maximum tolerated dose-feed / Water (g/kg)		Skin irritancy (Non vs. Irritant)	Skin sensitization (Weak vs. Strong)
	Value	Change rate (%)	Value	Change rate (%)		
ATZ	-1.608	-	0.004	-	0.999/I	0.789/S
D-5-P1	-5.103	-217.35	0.021	425.00	0.999/I	0.789/S
D-5-P2	-4.071	-153.17	0.157	3825.00	0.999/I	0.759/W

Continued Table S10. Human health risk assessment of D-5 microbial degradation products based on pharmacokinetic and toxicokinetic data

No.	Rodent carcinogenicity (Non vs. Carcinogen)			
	Male rat	Female rat	Male mouse	Female mouse
ATZ	0.442/Non	0.462/Non	0.602/Carc	0.476/Non
D-5-P1	0.561/Non	0.512/Non	0.435/Non	0.554/Non
D-5-P2	0.559/Non	0.525Non	0.597/Carc	0.561/Non

4. Materials and Methods

4.1 Characterization of herbicidal functionality properties, microbial degradability, and human lung cytotoxicity of S-THs - molecular docking method

S-THs target the D1 protein (D1-PSII) of photosynthetic system II [1] and inhibit weed photosynthesis, primarily through hydrolysis and metabolism of the triazine hy-drolase (TrzN) protein encoded by the trzN gene [2,3]. In addition, through cumu-lative effects on the lung and

respiratory system, S-TH molecules can cause damage to mammalian histone H2AX, which can be detrimental to human lung function [1,4].

4.3 Construction of a model for the comprehensive effects of herbicidal functionality properties, microbial degradability, and human lung cytotoxicity of S-THs - 3D-QSAR model

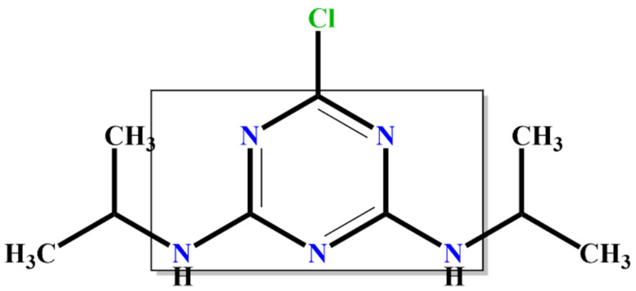

Figure S3. The structure and the common backbone of PRZ

Table S11. CoMSIA model evaluation methods and model evaluation criteria

Database	Validation	Parameters	Evaluation standard	References
Training	Cross Validation	Cross-validated	q^2 (>0.7)	[5]
		Optimal Principal Component	None	
	No-cross Validation	Standard Error of Estimate	SEE (lower is better)	[6]
		Non-cross-validated Correlation Coefficient	R^2 (close to 1 is batter)	
		Fischer Test Value	F (higher is better)	
Texting	External Validation	External Validation	r^2_{pred} (>0.6)	[7]
Overfitting Validation		——	$(R^2-q^2)/R^2$ (<30%)	[8]

4.5 Evaluation of the microbial degradability universality and toxicity of antioxidant systems in fish of S-THs substitutes - Molecular docking method

4.5.1 Evaluation of the microbial degradability universality of S-THs substitutes in the soil environment

It has been found that microbial N-isopropyl polyamide isopropylamino hydrolase (AtzC) in the soil environment has a degradation role and mechanism very similar to that of TrzN [9]. Further,

lignin peroxidase (LiP) and manganese peroxidase (MnP) of white-rot fungi can break down contaminants such as pesticides [10].

4.5.2 Evaluation of the toxicity of antioxidant systems in fish of S-THs substitutes in the aquatic environment

It has been demonstrated that introducing S-TH molecules into the aquatic environment can causes fish to produce large amounts of reactive oxygen species (ROS), resulting in oxidative stress. Although, superoxide dismutase (SOD) and catalase (CAT) can protect organisms from oxidative damage by scavenging ROS [11]. However, ATZ inhibits SOD and CAT activities in carp, Neotropical fish, and catfish, making them susceptible to oxidative damage, such as lipid peroxidation [12] and hepatotoxicity [13].

4.6 Screen of optimal field application schemes to promote the microbial degradation of S-THs substitutes in maize cropping fields - Taguchi orthogonal experiments, full factorial design of experiments, and molecular dynamics methods

Table S12. Nitrogen fertilizer and maize root secretion and their corresponding factors

Categories	Factors	Compounds	Chemical formulas	References
Fertilizer	A	Urea	CH_4N_2O	[14]
Organic acids	B	Glutaric acid	$C_5H_8O_4$	[15]
	C	Phthalic acid	$C_8H_6O_4$	
	D	Trichloroacetic acid	$C_2HCl_3O_2$	
	E	Benzoic acid	$C_7H_6O_2$	
Hydrocarbons	F	1-Octadecene	$C_{18}H_{36}$	
Aldehydes	G	E-14-Hexadecenal	$C_{16}H_{30}O$	
Ketones	H	Acetophenone	C_8H_8O	
Sugar	J	D-Glucose	$C_6H_{12}O_6$	[16]
	K	Arabinose	$C_5H_{10}O_5$	
	L	Fructose	$C_6H_{12}O_6$	
Organic acids	M	Fumaric acid	$C_4H_4O_4$	
	N	Oxalacetic acid	$C_4H_4O_5$	
	O	Citric acid	$C_6H_8O_7$	

Amino acids	P	Glutamic acid	$C_5H_9NO_4$
	Q	Aspartic acid	$C_4H_7NO_4$
	R	Alanine	$C_3H_7NO_2$
	S	Glycine	$C_2H_5NO_2$

4.7 Simulation of microbial degradation pathways for molecular of S-THs substitutes to S-THs - DFT and microbial degradation pathway simulation

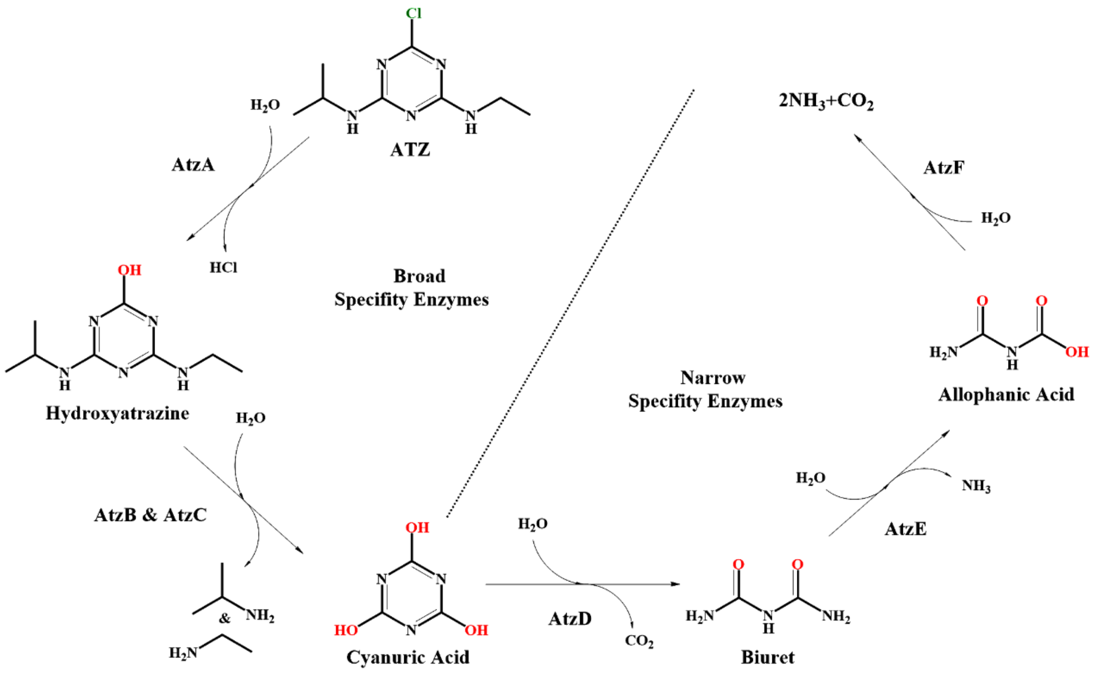

Figure S4. Microbial degradation pathway of ATZ

References

1. Liu, Q.Y. Research on the cytotoxicity and related mechanisms of herbicide prometrvn in vitro. Zhejiang University. **2017**.

2. Silva, V.P.; Moreira-Santos, M.; Mateus, C.; Teixeira, T.; Ribeiro, R.; Viegas, C.A. Evaluation of Arthrobacter aurescens Strain TC1 as bioaugmentation bacterium in soils contaminated with the herbicidal substance terbuthylazine. *PLoS One.* **2015**, 10, 1-15. DOI: 10.1371/journal.pone.0144978.

3. Cycon, M.; Mrozik, A.; Piotrowska-Seget, Z. Bioaugmentation as a strategy for the remediation of pesticide-polluted soil: A review. *Chemosphere* **2017**, 172, 52-71. DOI: 10.1016/j.chemosphere.2016.12.129.

4. Fernandez-Capetillo, O.; Lee, A.; Nussenzweig, M.; Nussenzweig, A. H2AX: The histone

guardian of the genome. *DNA Repair* **2004**, 3, 959-967. DOI: 10.1016/j.dnarep.2004.03.024.

5. Qu, R.J.; Liu, H.X.; Feng, M.B.; Yang, X.; Wang, Z.Y. Investigation on intramolecular hydrogen bond and some thermodynamic properties of polyhydroxylated anthraquinones. *J. Chem. Eng. Data.* **2012**, 57, 2442-2455. DOI: 10.1021/je300407g.
6. Yang, L.Z.; Liu, M. A double-activity (green algae toxicity and bacterial genotoxicity) 3D-QSAR model based on the comprehensive index method and its application in fluoroquinolones' modification. Int. J. Environ. Res. *Public Health* **2020**,17, 942. DOI: 10.3390/ijerph17030942.
7. Wang, X.L.; Gu, W.W.; Guo, E.M.; Cui, C.Y.; Li, Y. Assessment of long-range transport potential of polychlorinated Naphthalenes based on three-dimensional QSAR models. Environ. *Sci. Pollut. Res. Int.* **2017**, 24, 14802-14818. DOI: 10.1007/s11356-017-8967-8.
8. Veerasamy, R.; Rajak, H.; Jain, A.; Sivadasan, S.; Varghese, C.P.; Agrawal, R.K. Validation of QSAR models - strategies and importance. *Int. J. Drug Des. Discovery.* **2011**, 2, 511-519.
9. Karns, J.S. Gene sequence and properties of an s-triazine ring-cleavage enzyme from Pseudomonas sp. strain NRRLB-12227. *Appl. Environ. Microbiol.* **1999**, 65, 3512-3517. DOI: 10.1128/AEM.65.8.3512-3517.1999.
10. Asgher, M.; Bhatti, H.N.; Ashraf, M.; Legge, R.L. Recent developments in biodegradation of industrial pollutants by white rot fungi and their enzyme system. *Biodegradation* **2008**, 19, 771-783. DOI: 10.1007/s10532-008-9185-3.
11. Ru, S.G.; Wang, Y.; Zhang, X.N.; Yang, L.Q. Toxic effects of triazine herbicides on aquatic animals and their degradation method. *Periodical of Ocean University of China* **2022**, 52, 1-12. DOI: 10.16441/j.cnki.hdxb.20210183.
12. Santos, T.G.; Martinez, C.B.R. Atrazine promotes biochemical changes and DNA damage in a Neotropical fish species. *Chemosphere* **2012**, 89, 1118-1125. DOI: 10.1016/j.chemosphere.2012.05.096.
13. Mela, M.; Guiloski, I.C.; Doria, H.B.; Randi, M.A.F.; Ribeiro, C.A.D.; Pereira, L.; Maraschi, A.C.; Prodocimo, V.; Freire, C.A.; de Assis, H.C.S. Effects of the herbicide atrazine in neotropical catfish (Rhamdia quelen). Ecotoxicol. *Environ. Saf.* **2013**, 93, 13-21. DOI: 10.1016/j.ecoenv.2013.03.026.

14. Morris, T.F.; Murrell, T.S.; Beegle, D.B.; Camberato, J.J.; Ferguson, R.B.; Grove, J.; Ketterings, Q.; Kyveryga, P.M.; Laboski, C.A.M.; McGrath, J.M.; Meisinger, J.J.; Melkonian, J.; Moebius-Clune, B.N.; Nafziger, E.D.; Osmond, D.; Sawyer, J.E.; Scharf, P.C.; Smith, W.; Spargo, J.T.; van Es, H.M.; Yang, H.S. Strengths and limitations of nitrogen rate recommendations for corn and opportunities for improvement. *Agrronomy Journal* **2018**, 110, 1-37. DOI: 10.2134/agronj2017.02.0112.
15. Liu, X.W.; Wang, K.Q.; Zhao, Y.Y.; Duan, X.; Zhang, Y. Characteristics of maize root exudates at seedling stage and their response to rhizosphere soil enzyme activities under contour reverse-slope terrace. *Journal of Sichuan Agricultural University* **2021**, 39, 477-485. DOI: 10.16036/j.issn.1000-2650.2021.04.008.
16. Zhu, H.F.; Zhang, L.; Li, S.S.; Wang, Y.J.; Sun, S.R.; Chen, J.; Kou S.M.; Jin, Q.; Xiao, M. The rhizosphere and root exudates of maize seedlings drive plasmid mobilization in soil. *Applied Soil Ecology* **2018**, 124, 194-202. DOI: 10.1016/j.apsoil.2017.10.039.
